# Intrusive Growth of Phloem Fibers in Flax Stem: Integrated Analysis of miRNA and mRNA Expression Profiles

**DOI:** 10.3390/plants8020047

**Published:** 2019-02-19

**Authors:** Oleg Gorshkov, Tatyana Chernova, Natalia Mokshina, Natalia Gogoleva, Dmitry Suslov, Alexander Tkachenko, Tatyana Gorshkova

**Affiliations:** 1 Kazan Institute of Biochemistry and Biophysics, FRC Kazan Scientific Center of RAS, Lobachevsky Str., 2/31, 420111 Kazan, Russia; gorshkov@kibb.knc.ru (O.G.); chernova.t@mail.ru (T.C.); natalali@list.ru (N.M.); negogoleva@gmail.com (N.G.); 2 Laboratory of Extreme Biology, Institute of Fundamental Medicine and Biology, Kazan (Volga Region) Federal University, Kremlyovskaya Str., 18, 420021 Kazan, Russia; 3 Department of Plant Physiology and Biochemistry, Faculty of Biology, Saint Petersburg State University, Universiteskaya emb., 7/9, 199034 Saint Petersburg, Russia; d.suslov@spbu.ru; 4 Department of Genetics and Biotechnology, Faculty of Biology, Saint Petersburg State University, Universiteskaya emb., 7/9, 199034 Saint Petersburg, Russia; castorfiber@list.ru

**Keywords:** phloem fibers, intrusive growth, miRNA, transcriptome, laser microdissection, flax

## Abstract

Phloem fibers are important elements of plant architecture and the target product of many fiber crops. A key stage in fiber development is intrusive elongation, the mechanisms of which are largely unknown. Integrated analysis of miRNA and mRNA expression profiles in intrusivelygrowing fibers obtained by laser microdissection from flax (*Linum usitatissimum* L.) stem revealed all 124 known flax miRNA from 23 gene families and the potential targets of differentially expressed miRNAs. A comparison of the expression between phloem fibers at different developmental stages, and parenchyma and xylem tissues demonstrated that members of miR159, miR166, miR167, miR319, miR396 families were down-regulated in intrusively growing fibers. Some putative target genes of these miRNA families, such as those putatively encoding growth-regulating factors, an argonaute family protein, and a homeobox-leucine zipper family protein were up-regulated in elongating fibers. miR160, miR169, miR390, and miR394 showed increased expression. Changes in the expression levels of miRNAs and their target genes did not match expectations for the majority of predicted target genes. Taken together, poorly understood intrusive fiber elongation, the key process of phloem fiber development, was characterized from a miRNA-target point of view, giving new insights into its regulation.

## 1. Introduction

Phloem fibers are important elements of plant architecture by providing mechanical strength and support to a plant in general and to phloem in particular. These fibers are abundantly formed in the long but narrow stems of many fiber-crops. The specific mechanical properties of the phloem fibers are based on extraordinary length of their cells (many centimeters) and their very thick cell walls (up to 15 µm) [1]. Two key processes have a major impact on the specialization of the plant fibers: intrusive growth and cell wall thickening. They both are promising points for genetic manipulations to improve the yield and the quality of bast fibers [2]. In flax stems, these two stages of phloem fiber development are separated in time and space, allowing for the analysis of tissue and stage-specific components [2,3]. Flax stems contain only primary phloem fibers that originate from the procambium close to the stem apex [4,5]. Their development is more advanced towards the stem base. The complete cessation of intrusive growth and the onset of cell wall thickening are marked by the so-called “snap point” (SP) in flax phloem fibers, which is easy to find manually by stepwise increasing the effort required to break the stem [3].

Intrusive growth occurs when a growing cell increases in size faster than neighboring ones, thereby intruding between them [1,6,7]. Later, plant fiber specialization may advance by formation of tertiary cell wall, which is deposited after the primary and secondary cell walls in fibers of many species, including flax [8,9]. Tertiary cell walls are cellulose-enriched, and their cellulose microfibril orientation is close to axial. Entrapment of aggregated rhamnogalacturonan I molecules by laterally interacting cellulose microfibrills leads to tension of the latter and provides contractile properties to the fibers with tertiary cell walls [8,9].

In our previous work on flax plants based on RNA deep sequencing, we obtained transcript abundance profiles and identified a set of genes that are specifically activated at different stages of flax bast fiber development [10,11]. However, the mechanisms controlling both the intrusive elongation and the synthesis of tertiary cell wall, as well as the functioning of the fibers in the whole plant, are still poorly understood. 

The discovery of small RNAs (miRNA), a class of low molecular-weight, non-coding, regulatory RNAs of 19–24 nucleotides in size that act at the post-transcriptional level, added another level of complexity to the multi-level program for fine-tuning of gene expression [12,13]. miRNAs are able to regulate a whole range of biological processes, including developmental regulation, growth control, cell differentiation, and biotic and abiotic stresses. In fact, they may be the “master” non-protein regulators of plant and animal development [14,15,16]. The relative conservatism of miRNAs among plants of various taxonomic groups allows one to identify novel homologs of miRNA in different species using a database of already identified small RNA sequences and *in silico* methods [17,18,19,20].

124 miRNA sequences belonging to 23 families were identified in the flax genome, and more than a hundred of their target genes were predicted [18,21,22,23]. Prior miRNA research identified the roles of flax miRNAs and their potential targets in various stress responses, including nutrient excess or deficiency [24,25,26]. The samples used in these studies were complex mixtures of tissues that included many cell types and cells at differing developmental stages. Taking the advantage of the model system that has been well characterized in our previous works [3,5], we used high-throughput sequencing technology (Illumina) and bioinformatics tools to identify and analyze flax miRNAs and check the expression of their predicted targets in specific cell types and at certain stages of development. For the first time, miRNA and mRNA expression was analyzed during the poorly understood process of fiber intrusive elongation, giving new insights into the regulation of phloem fiber development.

## 2. Results

### 2.1. Deep-Sequencing of Flax Fiber Small RNAs

**Figure 1:**
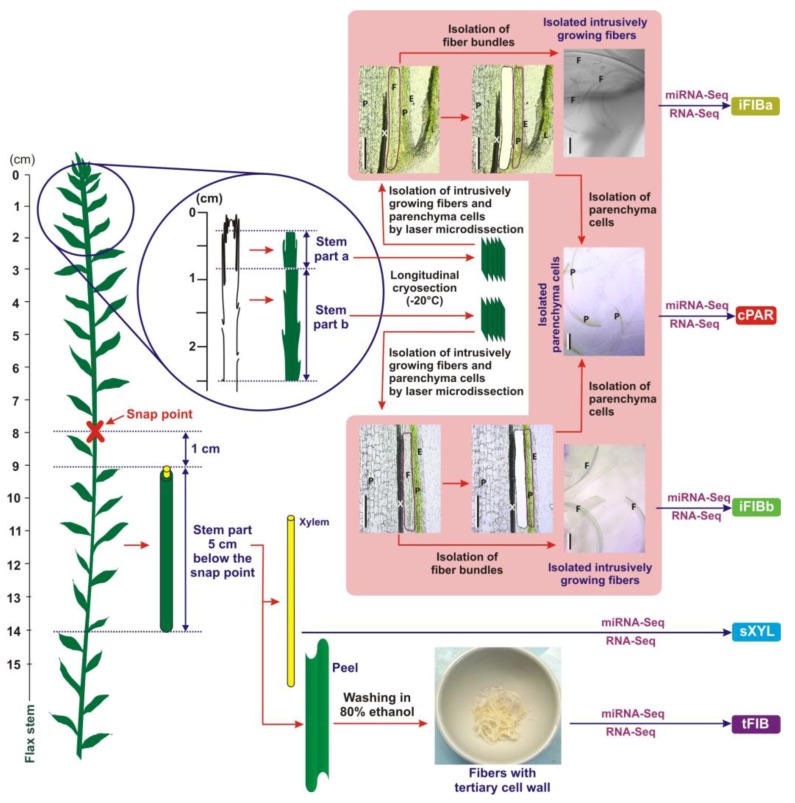
A scheme of plant material collection to obtain iFIBa, iFIBb, cPAR, sXYL and tFIB samples for subsequent RNA-Seq and miRNA-Seq analysis. Bundles of intrusively (i) growing fibers (the samples iFIBa and iFIBb) were isolated from longitudinal cryosections of the stem part a (0.3–0.8 cm from the stem apex) and b (0.8–2.5 cm from the stem apex) by laser microdissection. Cortex (c) parenchyma (cPAR) was isolated from longitudinal cryosections of the stem parts a and b by laser microdissection, and these two tissue fractions were combined. Fibers depositing tertiary (t) cell walls (tFIB) and xylem enriched in secondary (s) cell walls (sXYL) were sampled 1 cm below the snap point. E—epidermis, F—fiber bundles, L—leaf, P—parenchyma, and X—xylem; Bar—200 µm.

Ten individual libraries of small RNAs from two biological replicates of five different samples were analyzed by high throughput sequencing. The five samples included intrusively growing phloem fibers with only primary cell walls (iFIBa and iFIBb), symplastically growing cortex parenchyma (cPAR), phloem fibers at the stage of tertiary cell wall deposition (tFIB), and xylem (sXYL) that contained mainly cells with secondary cell walls (Figure 1). 

**Figure 2:**
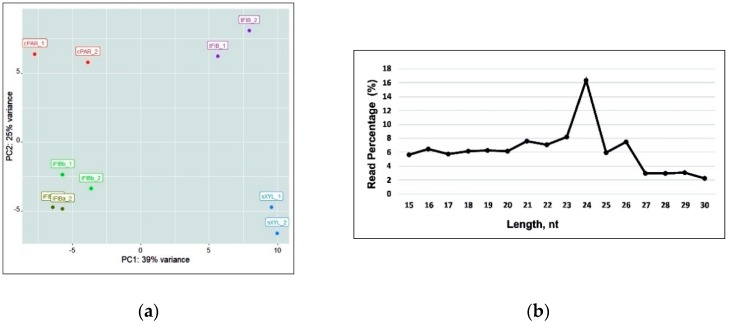
(**a**) The Principal Component Analysis (PCA) of the samples analyzed (including replicates) based on the lus-miR normalized expression pattern. (**b**) Size distribution of small RNA sequences in all of the libraries.

A total of 99,241,346 cleaned and filtered reads (79.98% of the raw miRNA reads) were used for further analysis (Table 1). miRNA expression levels from the 10 libraries datasets were visualized with the Principal Component Analysis (PCA) and analyzed for length distribution (Figure 2). PCA demonstrated that the two samples of intrusively growing fibers (iFIBa and iFIBb, differing in the stage of intrusive elongation; see a Section 2.5) were close to each other, but well separated from the other samples (cPAR, tFIB, sXYL); the variance of replicates within each sample was always much lower than that between the samples. The bulk of reads were 21–26 nt in length, with 24 nt being the most abundant group of small RNAs. Recently updated annotations of plant miRNA demonstrate that 23- or 24-nucleotide miRNAs are very rare and mostly characteristic of heterochromatic siRNAs [27]; nevertheless, the possibility that some plant species generate numerous 23- or 24-nucleotide microRNAs is not excluded [27]. The predominance of 24 nt read length is consistent with the distribution patterns of small RNAs in many plants [28,29,30,31,32], including flax [24].

### 2.2. Characterization of the Expressed Flax miRNAs

**Figure 3:**
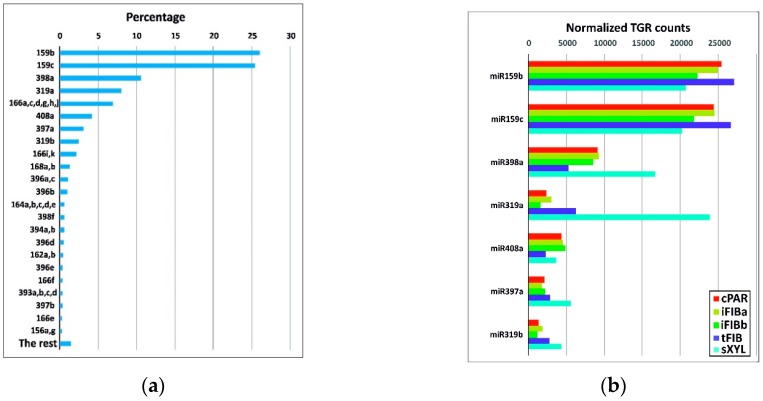
(**a**) The most abundant known miRNA families. The indicated miRNA flax families are represented by more than 1000 normalized TGR counts, and “The rest” have fewer than 1000 normalized TGR counts across all the samples as a whole. (**b**) Normalized TGR counts of the 5 most abundant miRNA families across the samples.

To identify the miRNAs expressed in the samples, the data from high-throughput small RNA sequencing were aligned against 124 flax miRNA precursor sequences. After estimating the expression values, representatives of the 23 known flax miRNA families were identified (Table S1). Representatives of 5 families were the most abundant in all the samples analyzed, with total gene reads (TGR) over 1000 in each sample, namely miR159, miR319, miR397, miR398, miR408 (Figure 3a).

miR159b and miR159c together accounted for over 50% of all detected miRNAs (Figure 3a). The miR159 family is highly conserved and widespread in plants, being expressed in the course of plant development and stress adaptation [17,24,33]. Expression of lus-miR159b and lus-miR159c was detected previously in various parts of flax plants, including stem, and suggested to be involved in the regulation and fine-tuning of the expression of some housekeeping genes [23].

The xylem samples were especially enriched in miR319a and miR398a compared with the other samples (Figure 3b). miR319 regulates transcription factors of the TCP family affecting multiple biological pathways: from hormone biosynthesis and signaling to cell proliferation and differentiation [34]. The three other families of most abundant miRNAs (miR408, miR398, and miR397) are Cu-miRNAs that are relatively conserved among plants and regulate a large number of Cu proteins [35].

### 2.3. Identification of Differentially Expressed miRNAs during Intrusive Elongation of Phloem Fibers in Flax Stems

**Figure 4:**
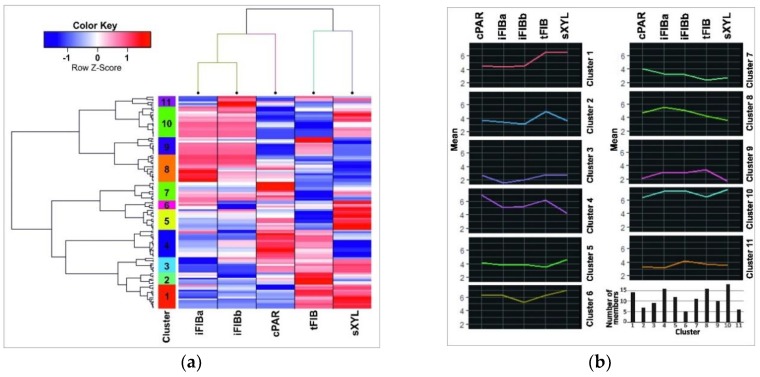
(**a**) A heatmap with dendrograms of hierarchical clustering of the data (the lus-miR subset and the samples). Columns represent samples, while rows represent 124 lus-miRs. The 11 clusters are coded by the vertical color bar. The Z-score is the number of standard deviations from the mean. The normalized expression value for each miRNA is depicted by color intensity, with red indicating up-regulated and blue indicating down-regulated lus-miRs. (**b**) Average expression profiles for each miRNA expression cluster and numbers of cluster members. An average cluster profile was calculated as the mean of normalized and scaled TGRs counts of all miRNAs in each cluster.

To figure out small RNAs that are especially abundant at certain developmental stages of flax phloem fiberes, the miRNA normalized TGR counts for all samples were used to generate a miR-expression heatmap and clustering (Figure 4). A dendrogram of miRNA expression derived from hierarchical clustergram analysis using the Pearson’s correlation was divided into 11 clusters (Figure 4a, the vertical color bar). 

A mean cluster profile for each cluster as the mean of TGR counts of all miRNAs in a cluster for each sample was calculated (Figure 4b). Clusters 1, 2, 4, and 6 contained miRNAs that were down-regulated in iFIB samples as compared to the other samples, whereas clusters 5, 7, 8, and 10 included up-regulated miRNAs.

To find out which miRNAs are differentially expressed during different stages of phloem fiber development (intrusive elongation and tertiary cell wall formation), pairwise comparisons between iFIBa vs. tFIB, and iFIBb vs. tFIB were performed. These comparisons revealed 19 down-regulated miRNAs during intrusive elongation that belonged to six families (miR156, 159, 166, 167, 319, 396) and 14 up-regulated miRNAs from five families (miR160, 169, 390, 394, and 399) (Table 2). A member of the miR396 family (miR396c) was down-regulated during intrusive fiber elongation in both a tissue- and stage-specific manner: the expression of this miRNA in both iFIBa and iFIBb was lower than that in the other tissues (cPAR and sXYL) and in the same tissue at the later stage of development (tFIB) (adjusted *p*-value (padj) < 0.05). Four different members of the miR396 family (a, b, d, e) were significantly down-regulated in intrusively growing fibers as compared with all other samples, except cPAR. In *Arabidopsis*, members of the miR396 family control cell proliferation and elongation [36]. Several members of miR166 were down-regulated in iFIB samples as compared to tFIB, cPAR, but not sXYL. As for miRNAs significantly up-regulated in iFIB, two members of miR394 had higher transcript abundance as compared to all other samples, except sXYL (Table 2).

### 2.4. Identification of miRNA Targets Up- and Down-Regulated during Intrusive Elongation of Phloem Fibers in Flax Stems

To understand the potential regulatory functions of miRNA that were differentially expressed during intrusive fiber growth, we used the psRNATarget web server that was developed to identify miRNA-target pairs by analyzing the complementary matching between small RNAs and targets and evaluating the accessibility of the target sites from the calculated value of unpaired energy (UPE) [37].

A total of 391,488,774 (97,52%) clean reads were obtained, of which about 88% reads were successfully mapped onto the flax reference genome [38]. To compare changes in gene expression between samples from different experiments, gene expression levels were obtained as normalized TGR using raw counts from HTSeq [39] and input into DESeq2 [40]. From 43,486 protein-coding genes of flax, 30,922 genes with normalized TGR values > 16 in at least one sample were selected for the analysis of differential expression in pairwise comparisons (Table S2). A total of 1942 up-regulated and 3765 down-regulated genes were identified in intrusively growing fibers when pairwise comparisons of iFIBa and iFIBb with tFIB were done (log_2_FC ≥ 2 or ≤ −2 with a cutoff padj < 0.05, Table S2). Among them, 1495 expressed targets (Table S2) for 33 miRNAs up- and down-regulated in iFIB as compared to tFIB (with a cutoff for fold change (FC) |log_2_FC| ≥ 2 and padj < 0.05) were predicted (Table 2).

The up-regulated targets for down-regulated miRNAs were enriched in homologs of *Arabidopsis* genes for transcription factors (SPL9, bZIP5, HB8, HB14, HB15, AP2/B3, and several GRFs) (Table 3). The most significantly down-regulated target genes included those for which *Arabidopsis* homologs were annotated as auxin-response factors, receptor kinases, transporters, and hormone-related (Table 4).

**Figure 5:**
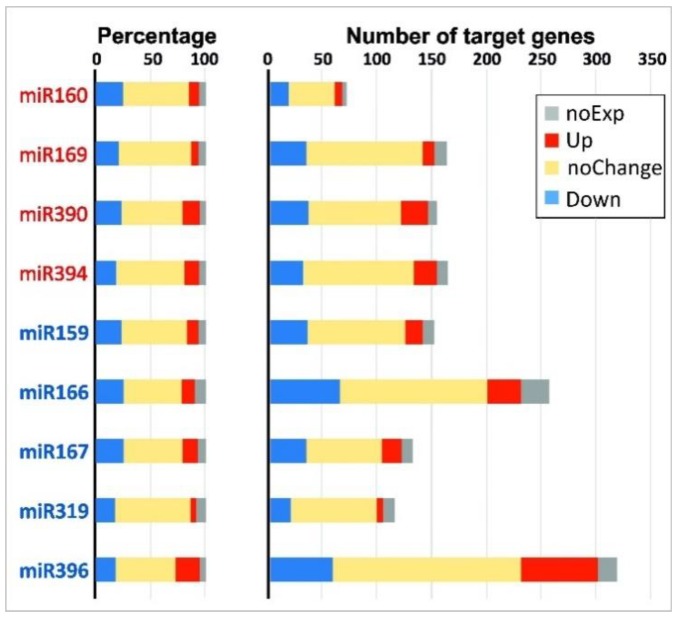
Proportion (**left**) and abundance (**right**) of differentially expressed target genes for up- (miR160, 169, 390 and 394, listed in red) and down-regulated (miR159, 166, 167, 319, 396, listed in blue) miRNA families for pairwise comparisons between iFIBa and tFIB (log_2_FC ≥ 1 or ≤−1). The up-regulated target genes are presented in red, the down-regulated - in blue, the non-expressed in the samples analyzed—in grey, and the genes without significant changes in transcription - in yellow.

For many combinations of miRNAs and their target genes, an opposite character of differential expression was observed, as expected from the general notion that miRNA marks the complementary mRNA for degradation [41]. For example, when comparing iFIBa and iFIBb versus tFIB, the expression of up- and down-regulated miRNAs was inversely related to the expression of 121 genes predicted as their targets (Table 3 and Table 4). However, these genes accounted for only a small portion of the predicted target sequences. The rest target genes were either not expressed in the samples analyzed (at least with the cutoff used), had no significant changes in expression, or changed their expression in the same direction as their targeting miRNAs. A comparison between iFIBs and tFIB samples revealed that the proportions of up-regulated (5–22%), down-regulated (17–25%), unchanged (53–69%), and non-expressed (5–10%) target mRNAs were rather similar for different miRNA families, independently of their behavior or numbers of their target genes (Figure 5). Notably, the average proportions of these groups of target mRNAs were similar between the up- and down-regulated miRNA families, and constituted 12 ± 4% and 13 ± 6% for the up-regulated, 22 ± 2% and 22 ± 4% for the down-regulated target genes, 58 ± 7% and 61 ± 5% for those with no changes in expression, and 6 ± 1% and 8 ± 2% for the non-expressed target genes, respectively.

### 2.5. Comparison of miRNA and mRNA Expression Profiles at the Two Substages of Fiber Intrusive Elongation

To identify genes and miRNAs, the expression of which differs at earlier and later stages of intrusive elongation, we subdivided the zone of intrusive elongation into two parts. The first part (a) was located above the second part (b) (Figure 1), so that (a) contained shorter fibers that were closer to the onset of intrusive elongation. Both samples had very similar mRNA expression patterns. In total, 557 genes were differentially expressed in iFIBa as compared to iFIBb (|log_2_FC| ≥ 1, padj < 0.05); among them, 114 genes were up-regulated in iFIBa (Table S3). An additional cutoff filtered out genes that were not differentially expressed between cortical parenchyma and intrusively growing fibers (|log_2_FC| ≥ 1 between iFIBa&b and cPAR), leaving 369 genes, of which 66 were up-regulated. 

Fourteen genes (Table 5), the mRNAs of which were enriched at the early stage of intrusive elongation in comparison with all other samples, were revealed. This most intriguing group contained genes for four putative homologs of transcription factors: a Myb domain protein 82 (Lus10038092), a basic helix-loop-helix (bHLH) DNA-binding superfamily protein (Lus10015909), an AT hook motif DNA-binding family protein (Lus10008497), and a growth-regulating factor 5 (GRF5, Lus10020352). In addition, the mRNA of *Arabidopsis* homolog encoding response regulator (ARR7, Lus10042938) was more abundant in iFIBa than in iFIBb and all other samples (Table 5). The largest difference in mRNA abundance between iFIBa and iFIBb was observed for a putative transducin/WD40 repeat-like protein (Lus10009510) and a dirigent protein 21 (Lus10016231), while the highest TGR value amongst the listed genes was found for a gene encoding putative acyl activating enzyme 12 (AAE12, Lus10020787). Of relevance to miRNA processing, a gene encoding a putative protein argonaute 7 (AGO7, Lus10037136) was up-regulated at the earlier stage of intrusive fiber elongation.

Amongst the 124 known flax miRNAs, only one—mi396b—was significantly down-regulated in iFIBa as compared to iFIBb (log_2_FC = −2.76, padj < 0.05). The predicted targets of this miRNA are growth-regulating factors 1 and 5 (GRF1, GRF5), as well as MAKR2 (a member of the membrane-associated kinase regulator gene family) (Table S2). GRF5 is also targeted by miR390 (up-regulated in iFIBa&b in comparison with the other samples, but without a significant difference between iFIBa and iFIBb), and by miR396a, miR396c, and miR396e (all down-regulated in iFIBa&b, as compared to the other samples, but with no significant difference between iFIBa and iFIBb).

## 3. Discussion

### 3.1. miRNAs and Their Predicted Targets That Are Important for Intrusive Growth of Phloem Fibers in Flax Stems

The role of small RNAs in the regulation of plant cell elongation is rather poorly characterized, especially in the case of intrusive growth. This is partly explained by difficulties in obtaining the sample for analysis. Intrusively growing phloem fibers are located deeply inside other stem tissues and are very prone to injury during their isolation procedure: they are already quite long, while still being surrounded by relatively weak primary cell walls only [1,5]. Intrusive growth of plant fibers is the key process of fiber development that determines the final length and width of each individual fiber, the number of cells in fiber bundles and their structure, as well as the strength of fiber connections within a bundle and with other surrounding cells [7,9,42]. The possibility to characterize this process at the cellular and molecular level is significant for fiber crops. The protocol we developed to isolate intrusively growing fibers using cryosectioning with subsequent laser microdissection will help achieve this characterization [11]. To figure out which miRNAs may be important at the stage of phloem fiber intrusive elongation, the expression patterns of all known flax miRNAs in different plant tissues, as well as in phloem fibers at different stages of development, were characterized.

Members of miR396, miR166, miR167, miR159, miR156, and miR319 families were significantly less abundant in phloem fibers during their intrusive growth as compared to the more advanced developmental stage when the thick tertiary cell wall is deposited. It is logical to assume that the up-regulation of these miRNAs at the advanced stage of fiber development could be required for the removal of transcripts which are essential for elongation, and, hence, completed their function. However further functional molecular genetic analysis is needed to check this hypothesis. It may be relevant for the growth regulating factors that were affected by miR396 (Table 3). However, the relationships between miRNAs and their target genes are not always straightforward: the expression of one gene might be regulated by more than one miRNA, and one miRNA is often able to regulate the expression of more than one target gene. For example, the low expression levels of miRNAs from the two families (miR166b and several representatives of miR396 family) in iFIB demonstrated a negative correlation with the high expression level of their predicted target Lus10012048. The putative *Arabidopsis* homolog of this gene encodes phragmoplast orienting kinesin 2 (Table 3), which was reported to be involved in the spatial control of cytokinesis [43]. The modulation of such control may be related to the development of multinuclearity, which takes place during intrusive elongation and is characteristic for primary phloem fibers [4,42].

Moreover, the expression levels of some predicted targets did not have an expected negative correlation with the expression level of the corresponding miRNA (Figure 5). This complex relation could be explained by several mechanisms. It is known that either sequence-specific degradation of target mRNAs or translational repression of the mRNA molecules occur depending on the degree of complementarity of the miRNA sequence with the target sequence [41]. Moreover, miRNAs can specifically activate translation under certain stress conditions, during development, and when cells exit the cell cycle and enter the quiescent stage [44]. The reversed role of miRNA may be coupled to the extent of base-pairing with the target mRNA, which is associated with the properties of the ribonucleoprotein complex [45,46]. In the case of miRNA effect on mRNA translation, the impact may not be visible at the level of transcriptome analysis. Additionally, the abundance of mRNA of a certain gene is the result of a combination of several factors, including activities of corresponding transcriptional regulators, an influence of more than one miRNA (Table 3), differences in the mechanisms, and the rate of mRNA decay [47]. Different combinations and hierarchy of such mechanisms may lead to the diversity of target mRNA abundance upon the specific change in miRNA level. Unidirectional changes in the expression of miRNAs and their target genes were also revealed in other studies, both in *Arabidopsis* [17] and flax [23]. The relative similarity in the proportion of various effects on the expression of genes targeted by different miRNA families (Figure 5) is to be explained in future studies.

### 3.2. Members of miR396 Family Are Down-Regulated in Intrusively Growing Phloem Fibers in Flax Stems

Several representatives of miR396 family were down-regulated during intrusive fiber elongation: their expression was the lowest as compared to the other samples (Table 2). This family is known to regulate the expression of genes for growth regulating factors (GRFs). So far, the importance of GRF was mainly reported for leaf development [48,49,50]. Overexpression of miR396 causes a drastic reduction in a cell number in *Arabidopsis*, while abolishing the regulation of GRF2 by miR396 promotes a moderate increase in organ size [49]. Moreover, miR396-targeted GRFs were required for *Arabidopsis* leaf adaxial–abaxial polarity and trichome formation, revealing the tight coordination of cell division and differentiation during leaf morphogenesis [50]. Probably, GRFs could also be involved in the regulation of fiber intrusive growth, since putative members of the GRF gene family were up-regulated in the apical part of flax stems. Although their expression subsequently decreased in fibers during intrusive elongation [11], it was rather tissue-specific because GRFs were expressed at a low level in parenchyma cells (Table 3). 

Lus10032452 and Lus10042952 were among other predicted targets of miR396; they both are homologous to the gene for ATP binding microtubule motor family protein HINKEL (Table 3). In *Arabidopsis*, HINKEL is expressed in microspores and developing pollen. Together with TETRASPORE, another microtubule motor family protein, it is responsible for cell plate expansion defects at cytokinesis during pollen mitosis I [51]. HINKEL gene was also up-regulated during Arabidopsis pollen tube growth, which is also characterized as intrusive growth [52].

In flax down-regulation of miR396a/c was reported under saline, alkaline, and saline-alkaline stress, while transgenic rice plants overexpressing miR396c were more sensitive to salt stress [53]. Osmotic stress was suggested to be highly probable in intrusively elongating fibers, since their enlargement is mainly achieved by increasing the vacuole through the accumulation of osmolytes and the resulting inward water flux [11]. Hence, down-regulation of this miRNA and the resulting decreased sensitivity to osmotic stress seems quite reasonable.

A potential target of miR396a and miR396c is a flax homolog of *Arabidopsis* AGO7 (up-regulated in intrusively growing fibers). These both miRs were down-regulated in intrusively growing fibers as compared to the other tissues. AGO7 is a highly specialized argonaute protein that regulates the auxin-signaling pathway via production of trans-acting siRNAs (ta-siRNAs) by interacting with miR390, which targets TAS3 transcripts for the biogenesis of ta-siRNAs [54,55]. TAS3 ta-siRNAs target several AUXIN RESPONSE FACTOR genes that are involved in the regulation of developmental timing and lateral organ development [56]. Genetic interaction was also shown to exists between the ta-siRNAs biogenesis machinery and the miR396 network during leaf development [57]. The increased expression of miR390 in iFIBs as compared to tFIB suggests a possible role during fiber elongation as well. 

### 3.3. Similar miRNA Families Are Related to Cell Elongation in Various Plant Tissues

Juvenile flax fibers had increased expression of miR160, miR169, miR390, miR394, miR399 compared to fibers with thickened cell walls (Table 2). In cotton seed hairs (trichomes with protrusive growth), miR167, miR396, and miR399 accumulation peaked during rapid elongation, the abundance of miR156 peaked at the end of cotton fiber elongation, and miR160 and miR390 were down-regulated [58]. In rice, miR169g, miR169p, and miR396c were highly accumulated in seedlings with active symplastic growth [59,60].

Several members of miR166 family were significantly down-regulated in phloem fibers during intrusive elongation as compared to the stage of cell wall thickening (Table 2). miR166 is an important regulator of stem apical meristem maintenance [61]. In *Arabidopsis*, argonaute 10 (AGO10) specifically sequesters miR166/165 to regulate shoot apical meristem (SAM) development [61]. Lus10039386 encoding a homolog of *Arabidopsis* AGO10 was up-regulated in intrusively growing fibers (Table S1). A similar situation was found for other targets of miR166-HD-ZIP III transcription factors required for the correct specification of the shoot and root apical meristems and procambial specification [61,62,63]. Putative members of HD-ZIP III group-homeobox-leucine zipper family proteins (ATHB8, 14 and 15) were also up-regulated in iFIBa&b (Table 3). Thus, miR166 down-regulation and the increased level of transcripts for some target genes could be the “vestige” from the SAM. However, the difference in expression of these genes with cortical parenchyma cells, which are located at the same stem level and are roughly at the same “age” as intrusively growing fibers, indicates that miR166 family and its targets can be essential during intrusive elongation. In flax, an increase in the expression level of miR166 in the apical part of the stem in comparison with the whole stem was shown previously [22]. However, the part of the flax stem bearing intrusively growing fibers was not considered in this prior study.

Despite some differences in miRNA expression in different samples of elongating plant cells (possibly caused by differences in the type of cell growth), miR396, miR166, miR167, miR169, miR160, miR156, miR319 gene families are likely important players in the process of elongation.

### 3.4. Genes Important at the Early Stage of Intrusive Elongation

Nothing is known about the mechanisms of intrusive growth initiation. To approach this problem, we compared mRNA and miRNA profiles in the fibers taken at the earlier and the later stage of elongation. Both samples had very similar gene expression patterns and rather limited number of genes and corresponding miRNAs that changed expression significantly (Table 5). Among them were genes, the homologs of which in *Arabidopsis* encode transducin/WD40, acyl activating enzyme 12 (AAE12), laccase 6, dirigent protein 21, glutamine dumper protein (GDU), transcription factors MYB82 and GRF5, and response regulator (ARR7). Members of the transducin/WD40 protein superfamily often function as molecular “hubs” mediating supramolecular interactions [64,65]. GDU are plant-specific membrane proteins that stimulate amino acid export by activating nonselective amino acid facilitators [66,67]. In *Arabidopsis*, MYB82 (AT5G52600) is expressed during trichome development [68]. GRF5 is a transcription activator that is strongly expressed in actively growing and developing tissues of Arabidopsis, including the shoot apical meristem [69]. An Arabidopsis homolog of this gene encodes two-component response regulator ARR7 that is involved in the His-to-Asp phosphorelay signal transduction system [70] and acts as a negative regulator of cytokinin signaling [71].

AAE12 has numerous functions and could be involved in the biosynthetic-oxidation of aromatic and cyclic plant hormones, such as jasmonic acid, auxin, and salicylic acid [72], in the accumulation of protective secondary metabolites, such as benzoyloxy glucosinolates [73]. Laccase 6 and dirigent protein 21 are usually associated with lignan and lignin biosynthesis [74,75], which does not take place in elongating cells. However, lac2 mutants showed compromised root elongation in *Arabidopsis* under PEG-induced dehydration conditions [76].

Other enzymes encoded by the up-regulated genes included homologs of *Arabidopsis* flavin-binding monooxygenase (probable indole-3-pyruvate monooxygenase YUCCA4 (YUC4, Lus10013125) that is involved in auxin biosynthesis and required for the formation of vascular tissues [77]), and heparan-alpha-glucosaminide N-acetyltransferase (Lus10021514).

Notably, fibers at the earlier stage of intrusive elongation were enriched in the transcripts of AGO7, which is involved in small RNA-mediated post-transcriptional gene silencing. It is the main component of the RNA-induced silencing complex (RISC) that binds to miRNAs for silencing the directed cleavage of homologous mRNAs to repress gene expression. AGO7 associates preferentially with miR390, which guides the cleavage of TAS3 precursor RNA [55]. AGO7 itself is a potential target for miR396a and miR396c. They both were down-regulated in intrusively growing fibers as compared to the other tissues. Thus, flax phloem fibers are enriched in several specific miRNAs at the beginning of intrusive elongation.

## 4. Materials and Methods

### 4.1. Plant Materials and Sample Preparation

Flax plants (*Linum usitatissimum* L., cultivar Mogilevsky from a collection of All-Russian Flax Research Institute, Torzhok) were grown in the open air under natural photoperiod in pots with 40 cm of soil and received daily watering. Samples for analysis were collected at the period of fast growth, when the stem height was 25–30 cm (40 days after sowing). The tissue sampling scheme for RNA-Seq analysis (Figure 1) was based on the characterized location of fibers in flax stems at different stages of development [3,5]. A set of samples consisted of: 1) intrusively growing phloem fibers with only primary cell walls (iFIBa and iFIBb) isolated from stem parts a (0.3–0.8 cm from the stem apex) and b (0.8–2.5 cm from the stem apex [11]) by laser microdissection; 2) symplastically growing cortex parenchyma (cPAR) isolated from the stem parts a and b by laser microdissection; 3) phloem fibers at the stage of tertiary cell wall deposition (tFIB) collected from a 5 cm long stem portion starting 1 cm below SP; and, 4) a xylem part (sXYL) that contained mainly cells with secondary cell wall, collected from the same stem portion as tFIB. The phloem fibers with tertiary cell walls were isolated from fiber-enriched stem peels by washing several times with 80 % ethanol in a mortar, while gently pressing with a pestle until other tissues and chlorophyll were removed (as described previously [78,79]). All collected samples were frozen in liquid nitrogen and stored at −80 °C until analysis.

### 4.2. Cryosectioning and Laser Microdissection

Flax stem pieces that included intrusively growing fibers and cortex parenchyma were cut off from the two parts of the stem (a and b) with a razor blade (Figure 1), then immediately frozen in liquid nitrogen and stored at −80 °C. Cryosectioning and fiber bundle isolation by laser microdissection followed the protocol [11]. Briefly, longitudinal stem cryosections (60-μm thick) were taken in a cryostat chamber (CM3050, Leica Microsystems, Wetzlar, Germany) at −20 °C and transferred onto a POL-membrane frame slide (Leica Microsystems). Microdissections were made by using a laser microdissection microscope (LMD7000, Leica Microsystems) at 10× magnification, the laser power of 37, the laser aperture of 11, and the laser speed of 4. Microdissected pieces were collected into the caps of 0.2-mL PCR tubes containing 20 μL of RNA lysis solution (RNAqueous-Micro RNA Isolation Kit, Ambion (Austin, TX, USA)) and stored at −80 °C. The identification of intrusively growing fibers was based on their localization in the stem (between the xylem and 5–6 cell layers of cortex parenchyma and epidermis), elongated cell shape, and their occurrence in bundles. Each final sample for RNA analysis (iFIBa, iFIBb, and cPAR) contained about 700 microdissected pieces obtained from 30 plants. 

### 4.3. RNA Isolation, Library Preparation and Sequencing

All samples were analyzed by next generation sequencing (NGS) in two independent biological replicates. Microdissected pieces from samples iFIBa, iFIBb, and cPAR were transferred from the PCR tube with RNA lysis solution into a microcentrifuge tube with 300 μL of the same lysis buffer for a silica column-based purification. Total RNA was extracted using an RNAqueous-Micro RNA Isolation Kit (Thermo Fisher Scientific, Waltham, MA, USA) according to the manufacturer’s instructions. Total RNA elution was performed with 2 × 10 μL elution buffer preheated to 95 °C. Total RNA from tFIB and sXYL (5 plants per sample) was isolated using Trizol combined with a mirVana miRNA Isolation Kit (Thermo Fisher Scientific, Lithuania), according to the manufacturer’s instructions. 

For all samples, any residual DNA was eliminated with a DNA-free DNA Removal kit (DNA-free DNA Removal kit, Waltham, MA, USA). RNA quantity and quality were analyzed by a Qubit fluorometer (Invitrogen, Carlsbad, CA, USA) and an Agilent 2100 Bioanalyzer (Agilent Technologies, Santa Clara, CA, USA). The RNA integrity index (RIN) was 7–8.4, which provides sufficient integrity for NGS. Total RNA of each sample was used to prepare both RNA-library and small RNA library.

cDNA libraries were prepared from the total RNA of iFIBa, iFIBb, cPAR, tFIB, and sXYL samples (up to 1 µg) with a NEBNext Ultra II Directional RNA Library Prep Kit (New England Biolabs, Ipswich, MA, USA) after selective depletion of ribosomal RNAs using a Ribo-Zero rRNA Removal Kit (Plant) (Illumina, San Diego, CA, USA), according to the manufacturer’s instructions. 

Libraries of small RNA were prepared with a NEBNext Multiplex Small RNA Library Prep Set for Illumina (New England Biolabs, Ipswich, MA, USA) according to the manufacturer’s protocol. Quality control of the libraries was performed using an Agilent 2100 Bioanalyzer (Agilent Technologies, Santa Clara, CA, USA). Further size selection was performed using Agencourt AMPure XP (Beckman Coulter, Indianapolis, Indiana, USA) to select for 143–149 bp fragments.

Sequencing was performed using a high-throughput HiSeq 2500 platform (Illumina, San Diego, CA, USA) in the mode of 60 bp single-end reads using HiSeq SR Cluster Kit v4 cBot and HiSeq SBS Kit v4 50 cycles kits (Illumina, San Diego, CA, USA). The sequences were deposited into the National Center for Biotechnology Information (NCBI) ([9], BioProject: PRJNA475325). 

### 4.4. Bioinformatic Analysis and Data Visualization

Adapter removal, quality trimming, and the removal of rRNAs, tRNAs, snRNAs and snoRNAs found in the RNACentral DB (a non-coding RNA sequence database) [80] were carried out using *BBDuk* utility of *BBTools*v 37.02 [81].

#### 4.4.1. RNA-seq Data Analysis

Clean reads for each sample were mapped onto the flax genome sequence scaffolds by using *HISAT2* v2.1.083 [82] with default parameters using the option --rna-strandness R. A reference genome sequence v. 1.0 *Linum usitatissimum* and an annotation as gff3 file were downloaded from Phytozome v.12 [38,83]. In addition to 43,484 protein-coding genes in the whole-genome assembly of flax, two CESA7 [78] genes were integrated into the existing annotation and numbered as *Lus10043485* and *Lus10043486*. Read count quantification for each gene was generated using *HTSeq-count* software [39] with default settings. For each gene total gene reads (TGR) was determined as the number of all reads that are mapped to this gene.

#### 4.4.2. miRNA-seq Data Analysis

After preprocessing raw data, we employed *cutadapt* [84] to remove any reads shorter than 15nt and longer than 30nt. Filtered small RNA reads from given libraries were aligned to 124 flax miRNA precursor sequences downloaded from DB RNACentral (116 out of these were catalogued in miRBase (release 22.1; [85])) using *bowtie2* v.2.2.9 [86] and then were counted to measure the expression of identified miRNAs by *samtools* v.1.3.1 [87].

#### 4.4.3. Differential Expression Analysis

The R package DESeq2 v.1.14.1 [40] was used to perform the differential expression analysis of both mRNA and miRNA from all samples using TGR counts generated for each sample, as described above. DESeq’s estimateSizeFactors and estimateDispersions functions (with default options) were used to obtain normalization factors for each sample and to normalize TGR counts. The normalization was applied to all samples simultaneously to ensure that the expression values were comparable across samples. In addition, for mRNA-seq, we pre-filtered the normalized TGR counts by removing the genes with a TGR < 16 across all samples according to the recommendations of the sequencing quality control project [88]. The resulting mRNA dataset consisted of 30,922 genes that were used for differential expression analysis. Principal component analysis (PCA), as implemented in DESeq2, was performed to investigate similarities between individual samples. Both mRNAs and miRNAs with log_2_FC ≥ 2 and adjusted *p*-value < 0.05 (determined according to [40]) were considered as up-regulated; with log_2_FC ≤ − 2 and adjusted *p*-value < 0.05- as down-regulated. For comparison of iFIBa and iFIBb, the threshold value was lowered to log_2_FC ≥ 1. In order to identify the features of gene expression in phloem fibers at the stage of intrusive growth compared to the other flax tissues or phloem fibers at the stage of deposition of the tertiary cell wall, the datasets from iFIBa and iFIBb samples were considered as biological replicates as input to DESeq2 with the name iFIBa&b.

#### 4.4.4. Hierarchical Clustering of Samples and miRNAs

Read counts after pre-filtering were normalized using the regularized-logarithm transformation or *rlog* [40], that stabilizes the variance across the mean, and the data become approximately homoscedastic [89]. The *rlog*-transformed values were used directly for computing the distances between samples, for making the PCA plot, and for clustering. The Euclidean distance and the Pearson correlation were used to group the samples and, respectively, to scale and group the genes using the R [90] and the *hclust* function. Ward.D2’s method was used in both cases. A dendrogram and a heatmap were generated using the R function *heatmap.2* in the *gplots* R package. The *cutree* function of *dendextend* R package was used to cut the dendrogram into clusters.

### 4.5. Computational Prediction of Flax miRNA Targets

The plant small RNA target analysis server (psRNATarget, [91]) with the “user-submitted small RNAs” option, along with the *Linum usitatissimum* sequences library (200 v1.0) from Phytozome v12 for a reference cDNA library and scoring schema V2 (2017 release), were used to identify the miRNA targets by finding complementary matches between miRNA and target transcripts [37].

## 5. Conclusions

The miRNAs and their target genes differentially expressed during intrusive elongation of phloem fibers have been identified in samples collected by laser microdissection from flax stems. miR396s and their targets (e.g., GROWTH-REGULATING FACTORs) are especially promising candidates for further study. Several members of miR396 family were down-regulated during intrusive fiber elongation in comparison with the fibers at a more advanced stage of development and different cell types, indicating both tissue- and stage-specific expression. The complex network that involves miR396, AGO7, and miR390 might play an important regulatory role during intrusive elongation. Members of miR166 family that are usually associated with the regulation of shoot apical meristem maintenance and vascular tissue differentiation, may also regulate intrusive growth of phloem fibers, since the specific character of expression for miR166 and its targets (AGO10 and HD-ZIP III) was revealed at this stage of fiber development. The expression levels of miRNAs and their target genes did not match expectations for many target genes.

So far, it is quite difficult to judge about the degree of universality of the regulation network recruiting miRNAs during different types of growth (symplastic, intrusive, protrusive), first of all, due to the scarcity of this kind of information and difficulties in individual cell type sampling. Probably, some miRNAs are more “general” and are involved in elongation of different tissues, acting, for example, on hormone-regulated genes, like miR160 and miR167, that play a role in auxin signaling [92]. However, the involvement of distinct miRNAs in the regulation of certain growth types cannot be excluded as well. Further studies are needed to highlight the universality or specificity of miRNA action in different types of growth.

## Figures and Tables

**Table 1:** Summary of reads in miRNA libraries.

	cPAR	iFIBa	iFIBb	tFIB	sXYL
Raw reads	25,949,391	27,817,347	26,770,365	22,233,088	21,301,402
Adapter removed	23,992,256	25,566,467	24,081,883	20,595,701	20,243,016
r-, t-, sn-, snoRNA removed	20,278,935	21,214,545	20,292,005	19,125,440	18,330,421
15–30 nt filtering	11,290,477	13,311,657	12,439,784	10,681,782	11,197,941
Reads that were too short, %	32.4	27.8	29.75	29.05	24.6
Reads that were too long, %	11.95	9.45	8.95	14.75	14.1
Filtered and cleaned reads, %	55.6	62.7	61.3	56.2	61.25

**Table 2:** The differentially expressed flax miRNAs the expression of which was significantly up- and down-regulated in intrusively growing flax fibers (the cutoff of comparison either iFIBa vs. tFIB, or iFIBb vs. tFIB has a log_2_FC value ≥2 or ≤−2 with padj < 0.05); FC; fold change.

Cluster.	lus_miR	iFIBa&b vs. cPAR, log_2_FC	padj	iFIBa vs. iFIBb, log_2_FC	padj	iFIBa vs. tFIB, log_2_FC	padj	iFIBb vs. tFIB, log_2_FC	padj	iFIBa&b vs. sXYL, log_2_FC	padj
	**DOWN-regulated miRNAs related to intrusive growth**										
1	miR396c	−2.00	3.2 × 10^−2^	−1.72	1.0	−9.04	3.1 × 10^−16^	−7.32	4.1 × 10^−19^	−7.40	1.2 × 10^−23^
	miR396d	−1.17	2.0 × 10^−1^	−1.86	1.0	−8.64	2.4 × 10^−19^	−6.78	1.1 × 10^−21^	−6.82	1.1 × 10^−24^
	miR396a	−1.08	2.0 × 10^−1^	−1.81	1.0	−7.86	1.1 × 10^−21^	−6.05	2.4 × 10^−20^	−6.12	2.7 × 10^−22^
	miR396b	−1.59	2.2 × 10^−1^	−2.76	7.5 × 10^−3^	−5.97	9.3 × 10^−17^	−3.21	1.9 × 10^−5^	−3.25	4.6 × 10^−3^
	miR396e	−1.40	2.9 × 10^−1^	−1.93	1.0	−4.43	1.3 × 10^−6^	−2.50	1.0 × 10^−2^	−3.64	1.3 × 10^−3^
	miR156a	−0.32	8.2 × 10^−1^	0.10	1.0	−3.24	5.8 × 10^−4^	−3.34	6.3 × 10^−4^	−2.47	1.3 × 10^−3^
	miR167i	1.40	2.8 × 10^−1^	0.03	1.0	−2.47	2.9 × 10^−2^	−2.50	2.8 × 10^−2^	−1.69	5.3 × 10^−2^
	miR159a	3.74	7.6 × 10^−2^	2.18	1.0	−2.12	9.2 × 10^−2^	−4.30	9.1 × 10^−3^	−3.97	2.9 × 10^−5^
2	miR166f	−2.47	1.8 × 10^−4^	−0.90	1.0	−4.22	3.7 × 10^−9^	−3.32	7.8 × 10^−6^	−0.50	5.3 × 10^−1^
	miR166e	−1.90	3.5 × 10^−3^	−0.49	1.0	−3.60	4.8 × 10^−7^	−3.11	2.1 × 10^−5^	−0.47	5.4 × 10^−1^
4	miR166a	−3.75	5.0 × 10^−12^	−0.70	1.0	−2.71	2.2 × 10^−5^	−2.00	3.5 × 10^−3^	2.37	2.9 × 10^−5^
	miR166b	−2.52	1.6 × 10^−4^	−0.58	1.0	−2.71	7.1 × 10^−4^	−2.12	1.0 × 10^−2^	1.69	1.7 × 10^−2^
	miR166c	−3.54	2.5 × 10^−11^	−0.41	1.0	−2.41	2.4 × 10^−4^	−2.00	4.1 × 10^−3^	2.43	1.5 × 10^−5^
	miR166h	−3.34	1.2 × 10^−10^	−0.52	1.0	−2.39	1.9 × 10^−4^	−1.86	5.3 × 10^−3^	2.32	2.5 × 10^−5^
	miR166k	−3.63	1.3 × 10^−10^	−0.72	1.0	−2.35	5.8 × 10^−4^	−1.63	2.6 × 10^−2^	2.88	1.7 × 10^−6^
	miR166d	−3.72	5.0 × 10^−12^	−0.68	1.0	−2.35	2.4 × 10^−4^	−1.67	1.2 × 10^−2^	2.35	2.8 × 10^−5^
	miR166j	−3.63	6.0 × 10^−11^	−0.69	1.0	−2.19	1.1 × 10^−3^	−1.50	3.7 × 10^−2^	2.76	2.5 × 10^−6^
	miR166g	−3.32	3.7 × 10^−10^	−0.59	1.0	−2.08	1.4 × 10^−3^	−1.48	3.3 × 10^−2^	2.77	8.5 × 10^−7^
6	miR319a	−0.01	9.9 × 10^−1^	0.97	1.0	−1.04	9.6 × 10^−2^	−2.01	6.3 × 10^−4^	−3.39	6.5 × 10^−11^
	**UP-regulated miRNAs related to intrusive growth**										
5	miR390a	−0.55	4.9 × 10^−1^	0.05	1.0	2.03	1.5 × 10^−2^	1.98	2.0 × 10^−2^	−1.19	5.3 × 10^−2^
	miR390c	−1.53	3.2 × 10^−2^	−0.26	1.0	2.39	1.0 × 10^−2^	2.65	4.8 × 10^−3^	−1.58	1.6 × 10^−2^
7	miR390b	−1.71	2.2 × 10^−2^	0.58	1.0	2.58	5.7 × 10^−3^	2.01	4.6 × 10^−2^	−1.55	2.4 × 10^−2^
	miR169k	0.08	9.4 × 10^−1^	0.19	1.0	3.44	1.7 × 10^−3^	3.25	5.1 × 10^−3^	3.49	7.1 × 10^−5^
8	miR399d	1.56	1.8 × 10^−1^	0.70	1.0	2.55	3.0 × 10^−2^	1.86	1.3 × 10^−1^	3.11	1.0 × 10^−3^
	miR160h	1.71	1.4 × 10^−1^	0.10	1.0	2.58	4.2 × 10^−2^	2.48	5.1 × 10^−2^	3.15	1.2 × 10^−3^
	miR160j	1.16	3.6 × 10^−1^	0.51	1.0	2.97	2.9 × 10^−2^	2.45	7.1 × 10^−2^	4.15	2.9 × 10^−4^
	miR160a	1.31	2.2 × 10^−1^	0.60	1.0	3.06	8.1 × 10^−3^	2.46	4.4 × 10^−2^	4.05	2.9 × 10^−5^
	miR160i	0.97	4.9 × 10^−1^	1.06	1.0	3.18	2.6 × 10^−2^	2.12	1.6 × 10^−1^	4.06	8.1 × 10^−4^
	miR160d	0.47	7.1 × 10^−1^	1.15	1.0	3.46	5.6 × 10^−4^	2.31	3.7 × 10^−2^	3.76	2.6 × 10^−5^
	miR160b	0.58	6.6 × 10^−1^	0.34	1.0	3.51	3.6 × 10^−3^	3.18	1.1 × 10^−2^	4.46	1.8 × 10^−5^
	miR169e	1.98	1.0 × 10^−1^	0.13	1.0	3.63	5.7 × 10^−3^	3.51	1.0 × 10^−2^	3.23	1.2 × 10^−3^
10	miR394b	2.78	1.8 × 10^−6^	0.46	1.0	2.74	2.3 × 10^−5^	2.28	7.9 × 10^−4^	−1.97	3.3 × 10^−4^
	miR394a	2.68	3.9 × 10^−4^	0.03	1.0	2.96	3.9 × 10^−4^	2.93	6.4 × 10^−4^	−1.64	1.7 × 10^−2^

**Table 3:** Intrusive growth-related down-regulated lus-miR families and their up-regulated target genes identified from the comparison of phloem fibers at the stages of intrusive elongation and cell wall thickening (a cutoff of comparison either iFIBa vs. tFIB, or iFIBb vs. tFIB has a log_2_FC value ≥2 or ≤−2 with padj < 0.05); FC; fold change.

Family of miR	Member of lus-miR Family	Transcript ID	At Homolog	At Symbol	Description	iFIBa vs. tFIB, log_2_FC	padj	iFIBb vs. tFIB, log_2_FC	padj
**miR156**	a	Lus10021034	AT2G42200	SPL9	squamosa promoter binding protein-like 9	4.43	3.68 × 10^−89^	4.67	5.11 × 10^−99^
	a	Lus10002430	AT3G62390	TBL6	TRICHOME BIREFRINGENCE-LIKE 6	3.34	8.55 × 10^−12^	3.12	2.46 × 10^−10^
	a	Lus10023818	AT2G42200	SPL9	squamosa promoter binding protein-like 9	2.87	1.04 × 10^−10^	2.85	1.60 × 10^−10^
	a	Lus10024555	AT4G12110	SMO1	sterol-4alpha-methyl oxidase 1-1	2.53	8.25 × 10^−33^	2.61	1.20 × 10^−34^
	a	Lus10012020	AT2G42200	SPL9	squamosa promoter binding protein-like 9	2.44	6.94 × 10^−19^	2.25	4.82 × 10^−16^
	a	Lus10021141	AT1G69170		Squamosa promoter-binding protein-like (SBP domain) transcription factor family protein	2.08	2.14 × 10^−10^	1.46	1.87 × 10^−5^
	a	Lus10007726	AT3G49760	bZIP5	basic leucine-zipper 5	1.97	6.20 × 10^−3^	3.23	2.10 × 10^−6^
**miR159**	a	Lus10027321	AT5G47560	TDT	tonoplast dicarboxylate transporter	4.99	5.39 × 10^−3^	6.78	9.58 × 10^−5^
	a	Lus10031827	AT5G12930			3.86	1.89 × 10^−19^	3.31	2.65 × 10^−14^
	a	Lus10041729	AT5G25620	YUC6	Flavin-binding monooxygenase family protein	3.8	2.85 × 10^−2^	4.62	6.86 × 10^−3^
	a	Lus10016550	AT5G07900		Mitochondrial transcription termination factor family protein	3.23	1.81 × 10^−7^	2.77	1.38 × 10^−5^
	a	Lus10034196	AT3G11920		glutaredoxin-related	3.06	1.60 × 10^−8^	2.18	1.17 × 10^−4^
	a	Lus10031256	AT5G12930			3.04	2.98 × 10^−6^	2.61	1.02 × 10^−4^
	a	Lus10019870	AT3G19184		AP2/B3-like transcriptional factor family protein	2.11	5.70 × 10^−4^	1.43	3.14 × 10^−2^
**miR166**	a,b,c,d,e,f,g,h,i,j,k	Lus10029117	AT1G17100		SOUL hem × 10-binding family protein	7.05	6.17 × 10^−5^	6.78	1.43 × 10^−4^
	b	Lus10022678	AT3G51740	IMK2	inflorescence meristem receptor-like kinase 2	3.04	3.50 × 10^−12^	2.87	8.73 × 10^−11^
	a,c,d,e,f,g,h,i,j,k	Lus10030381	AT2G45850		AT hook motif DNA-binding family protein	2.87	4.96 × 10^−33^	2.56	4.37 × 10^−26^
	a,b,c,d,e,f,g,h,i,j,k	Lus10027060	AT3G59680			2.67	4.26 × 10^−4^	3.39	4.96 × 10^−6^
	a,b,c,d,e,f,g,h,i,j,k	Lus10023357	AT2G34710	HB14,PHB	Homeobox-leucine zipper family protein/lipid-binding START domain-containing protein	2.42	1.15 × 10^−39^	2.13	8.75 × 10^−31^
	a,b,c,d,e,f,g,h,i,j,k	Lus10037568	AT1G52150	HB15,CNA,ICU4	Homeobox-leucine zipper family protein/lipid-binding START domain-containing protein	2.38	8.48 × 10^−55^	2.01	7.92 × 10^−39^
	a,b,c,d,e,f,g,h,i,j,k	Lus10038449	AT2G34710	HB14,PHB	Homeobox-leucine zipper family protein/lipid-binding START domain-containing protein	2.3	3.80 × 10^−39^	2.09	4.33 × 10^−32^
	a,c,d,e,f,g,h,i,j,k	Lus10037696	AT5G63950	CHR24	chromatin remodeling 24	2.21	2.98 × 10^−20^	1.9	4.84 × 10^−15^
	a,b,c,d,e,f,g,h,i,j,k	Lus10011426	AT4G32880	HB8	homeobox gene 8	2.13	1.14 × 10^−32^	1.79	4.14 × 10^−23^
	a,b,c,d,e,f,g,h,i,j,k	Lus10011616	AT4G32880	HB8	homeobox gene 8	2.1	5.98 × 10^−14^	1.94	5.89 × 10^−12^
	a,b,c,d,e,f,g,h,i,j,k	Lus10025223	AT3G56370		Leucine-rich repeat protein kinase family protein	2	1.31 × 10^−18^	2.1	3.33 × 10^−20^
**miR166 miR396**	b a,b,c,e	Lus10012048	AT3G19050	POK2	phragmoplast orienting kinesin 2	2.49	1.92 × 10^−30^	2.48	4.58 × 10^−30^
**miR167**	i	Lus10026283	AT2G21180			4.41	4.80 × 10^−2^	4.7	3.59 × 10^−2^
	i	Lus10006347	AT5G12080	MSL10	mechanosensitive channel of small conductance-like 10	2.68	6.14 × 10^−4^	2.8	3.62 × 10^−4^
	i	Lus10029912	AT3G05330	ATTAN	cyclin family	2.28	4.39 × 10^−10^	2.46	1.62 × 10^−11^
	i	Lus10020804	AT5G37020	ARF8	auxin response factor 8	2.19	1.21 × 10^−20^	1.98	5.94 × 10^−17^
	i	Lus10038019	AT2G46980			2.07	8.42 × 10^−6^	2.26	1.10 × 10^−6^
	i	Lus10031354	AT5G37020	ARF8	auxin response factor 8	1.96	8.84 × 10^−53^	2.02	8.09 × 10^−56^
	i	Lus10002960	AT5G12080	MSL10	mechanosensitive channel of small conductance-like 10	0.88	4.13 × 10^−1^	2.03	3.15 × 10^−2^
**miR319**	a	Lus10041994	AT5G52450		MATE efflux family protein	2.9	2.27 × 10^−25^	2.26	1.44 × 10^−15^
	a	Lus10004295	AT5G49160	DMT1,MET1,MET2	methyltransferase 1	2.04	5.75 × 10^−22^	1.99	8.43 × 10^−21^
**miR396**	a,b,c,e	Lus10038002	AT2G45480	GRF9	growth-regulating factor 9	7.09	7.27 × 10^−4^	4.46	5.41 × 10^−2^
	a,b,c,e	Lus10019275	AT2G22840	GRF1	growth-regulating factor 1	5.84	2.41 × 10^−12^	3.53	4.90 × 10^−5^
	a,b,c,e	Lus10019274	AT2G22840	GRF1	growth-regulating factor 1	5.14	1.60 × 10^−23^	2.78	2.52 × 10^−7^
	a,b,c,e	Lus10000473	AT3G13960	GRF5	growth-regulating factor 5	4.75	6.53 × 10^−5^	3.55	4.21 × 10^−3^
	a,b,c,e	Lus10011559	AT2G22840	GRF1	growth-regulating factor 1	4.5	2.91 × 10^−6^	2.11	4.80 × 10^−2^
	a,b,c,e	Lus10009234	AT2G45480	GRF9	growth-regulating factor 9	4.06	2.83 × 10^−2^	3.6	6.19 × 10^−2^
	a,c	Lus10042952	AT1G18370	HIK,NACK1	ATP binding microtubule motor family protein	3.49	9.07 × 10^−39^	3.32	7.30 × 10^−35^
	a,b,c,e	Lus10011558	AT2G22840	GRF1	growth-regulating factor 1	3.48	4.32 × 10^−12^	0.67	2.90 × 10^−1^
	a,b,c,e	Lus10031717	AT5G62360		Plant invertase/pectin methylesterase inhibitor superfamily protein	3.46	8.90 × 10^−34^	3.68	4.89 × 10^−38^
	a,c	Lus10032452	AT1G18370	HIK,NACK1	ATP binding microtubule motor family protein	3.19	3.52 × 10^−41^	3.17	1.55 × 10^−40^
	a,b,c,e	Lus10028754	AT1G64080			3.04	6.45 × 10^−6^	0.82	3.35 × 10^−1^
	a,c	Lus10004012	AT3G61740	ATX3,SDG14	SET domain protein 14	2.9	2.28 × 10^−7^	3.22	7.72 × 10^−9^
	d	Lus10024639	AT4G23440		Disease resistance protein (TIR-NBS class)	2.76	2.89 × 10^−4^	1.62	5.34 × 10^−2^
	d	Lus10039159	AT1G33500			2.71	3.25 × 10^−2^	2.57	4.91 × 10^−2^
	a,b,c,e	Lus10010519	AT3G06030	MAPKKK12,NP3	NPK1-related protein kinase 3	2.69	9.82 × 10^−26^	2.61	4.96 × 10^−24^
	b,e	Lus10006983	AT5G57655		xylose isomerase family protein	2.59	1.22 × 10^−42^	2.01	7.05 × 10^−26^
	a,b,c,e	Lus10027933	AT3G19050	POK2	phragmoplast orienting kinesin 2	2.57	2.29 × 10^−32^	2.61	3.04 × 10^−33^
	d	Lus10018093	AT3G48210			2.47	1.92 × 10^−19^	2.38	5.86 × 10^−18^
	a,b,c,e	Lus10033441	AT3G13960	GRF5	growth-regulating factor 5	2.46	7.44 × 10^−5^	1.56	1.92 × 10^−2^
	a,b,c,e	Lus10033236	AT4G24150	GRF8	growth-regulating factor 8	2.25	8.03 × 10^−4^	1.1	1.56 × 10^−1^
	a,b,c,e	Lus10008268	AT4G24150	GRF8	growth-regulating factor 8	2.24	1.04 × 10^−3^	1.05	1.80 × 10^−1^
	a,b,c,e	Lus10009533	AT3G13960	GRF5	growth-regulating factor 5	2.21	2.19 × 10^−14^	1.23	7.23 × 10^−5^
	b,e	Lus10043134	AT1G04730	CTF18	P-loop containing nucleoside triphosphate hydrolases superfamily protein	2.15	9.94 × 10^−21^	2.2	1.37 × 10^−21^
	d	Lus10004364	AT4G16820	PLA-I{beta]2	alpha/beta-Hydrolases superfamily protein	2.12	3.86 × 10^−15^	1.28	7.12 × 10^−6^
	a,c	Lus10037136	AT1G69440	AGO7,ZIP	Argonaute family protein	2.1	1.60 × 10^−10^	0.27	5.49 × 10^−1^
	d	Lus10034335	AT1G25400			1.75	1.72 × 10^−4^	2.09	5.30 × 10^−6^

**Table 4:** Intrusive growth-related up-regulated lus-miR families and their down-regulated target genes identified from the comparison of phloem fibers at the stages of intrusive elongation and cell wall thickening (a cutoff of comparison either iFIBa vs. tFIB, or iFIBb vs. tFIB has a log_2_FC value ≥2 or ≤−2 with padj < 0.05).

Family of miR	Member of lus-miR Family	Transcript ID	At Homolog	At Symbol	Description	iFIBa vs. tFIB, log_2_FC	padj	iFIBb vs. tFIB, log_2_FC	padj
miR160	a,b,d,h,i,j	Lus10002356	AT4G13830	J20	DNAJ-like 20	−5.5	9.25 × 10^−3^	−0.69	7.18 × 10^−1^
	a,b,d,h,i,j	Lus10016090	AT2G28350	ARF10	auxin response factor 10	−4.05	1.93 × 10^−21^	−2.92	1.27 × 10^−14^
	a,b,d,h,i,j	Lus10018087	AT3G48360	BT2	BTB and TAZ domain protein 2	−3.39	2.08 × 10^−21^	−3.9	9.03 × 10^−27^
	a,b,d,h,i,j	Lus10026510	AT4G30080	ARF16	auxin response factor 16	−2.98	6.02 × 10^−11^	−2.5	4.18 × 10^−8^
	a,b,d,h,i,j	Lus10019940	AT4G30080	ARF16	auxin response factor 16	−2.79	5.29 × 10^−9^	−3.1	1.54 × 10^−9^
	a,b,d,h,i,j	Lus10021467	AT4G30080	ARF16	auxin response factor 16	−2.71	2.06 × 10^−12^	−1.74	4.11 × 10^−6^
	a,b,d,h,i,j	Lus10007948	AT4G30190	HA2,PMA2	H(+)-ATPase 2	−2.69	1.23 × 10^−48^	−2.36	1.64 × 10^−37^
	a,b,d,h,i,j	Lus10010263	AT4G37250		Leucine-rich repeat protein kinase family protein	−2.11	5.48 × 10^−25^	−2.04	3.02 × 10^−23^
	a,b,d,h,i,j	Lus10042082	AT3G48360	BT2	BTB and TAZ domain protein 2	−1.87	1.05 × 10^−12^	−2.3	4.72 × 10^−18^
miR169	e,k	Lus10011003	AT5G02890		HXXXD-type acyl-transferase family protein	−10.21	5.23 × 10^−10^	−6.55	1.78 × 10^−11^
	k	Lus10008780	AT3G01500	ATSABP3,CA1	carbonic anhydrase 1	−7.78	2.99 × 10^−4^	−2.33	2.81 × 10^−1^
	k	Lus10014812	AT4G27290		S-locus lectin protein kinase family protein	−6.83	7.34 × 10^−5^	−4.19	2.06 × 10^−3^
	e	Lus10028782	AT4G21380	RK3	receptor kinase 3	−6.02	3.94 × 10^−17^	−6.3	5.02 × 10^−15^
	k	Lus10022235	AT3G01500	SABP3,CA1	carbonic anhydrase 1	−5.69	5.27 × 10^−9^	−3.56	4.05 × 10^−4^
	k	Lus10031671	AT5G07200	GA20OX3,YAP169	gibberellin 20-oxidase 3	−5.53	1.57 × 10^−4^	−7.74	2.26 × 10^−5^
	k	Lus10028494	AT1G66880		Protein kinase superfamily protein	−5.17	3.00 × 10^−10^	−5.57	2.82 × 10^−10^
	k	Lus10007724				−4.43	1.20 × 10^−14^	−2.66	1.11 × 10^−7^
	e	Lus10023139	AT3G62700	MRP10	multidrug resistance-associated protein 10	−4.02	2.22 × 10^−36^	−2.72	9.29 × 10^−19^
	e	Lus10019657	AT2G23450		Protein kinase superfamily protein	−3.79	8.29 × 10^−30^	−2.9	1.44 × 10^−19^
	e	Lus10000741	AT2G23450		Protein kinase superfamily protein	−3.44	2.60 × 10^−22^	−2.96	3.16 × 10^−17^
	e	Lus10016493	AT1G07430	HAI2	highly ABA-induced PP2C gene 2	−2.88	2.63 × 10^−8^	−3.33	7.92 × 10^−10^
	e	Lus10040231	AT2G39920		HAD superfamily, subfamily IIIB acid phosphatase	−2.82	2.71 × 10^−3^	−4.86	8.06 × 10^−5^
	e	Lus10033665	AT1G03790	SOM	Zinc finger C-x8-C-x5-C-x3-H type family protein	−2.52	2.05 × 10^−2^	−8.55	3.52 × 10^−6^
	e,k	Lus10001275	AT1G76680	OPR1	12-oxophytodienoate reductase 1	−2.45	5.20 × 10^−2^	−2.98	2.48 × 10^−2^
	k	Lus10040311	AT2G39510		nodulin MtN21 /EamA-like transporter family protein	−2.45	1.01 × 10^−3^	−2.6	5.16 × 10^−4^
	e,k	Lus10009473	AT1G76690	OPR2	12-oxophytodienoate reductase 2	−2.37	4.46 × 10^−13^	−2.35	1.09 × 10^−12^
	k	Lus10030400	AT5G60900	RLK1	receptor-like protein kinase 1	−2.36	1.32 × 10^−3^	−1.86	1.39 × 10^−2^
	e	Lus10002765	AT4G18950		Integrin-linked protein kinase family	−2.2	2.88 × 10^−3^	−3.61	9.09 × 10^−6^
	e	Lus10011498	AT2G47800	EST3,MRP4	multidrug resistance-associated protein 4	−2.11	1.36 × 10^−19^	−1.12	3.07 × 10^−6^
	k	Lus10039629	AT4G37930	SHM1,STM	serine transhydroxymethyltransferase 1	−2.11	1.27 × 10^−9^	−1.44	6.52 × 10^−5^
	e	Lus10029265	AT4G18820		AAA-type ATPase family protein	−2.02	4.10 × 10^−9^	−1.63	2.77 × 10^−6^
	e	Lus10031828	AT4G12070			−0.72	2.86 × 10^−1^	−2.69	1.35 × 10^−4^
miR390	a,b,c	Lus10005017	AT4G08850		Leucine-rich repeat receptor-like protein kinase family protein	−11.56	2.46 × 10^−13^	−11.36	7.56 × 10^−13^
	a,b,c	Lus10005042	AT5G01360	TBL3	Plant protein of unknown function (DUF828)	−9.48	6.10 × 10^−58^	−8.76	3.31 × 10^−67^
	a,b,c	Lus10018911	AT3G24240		Leucine-rich repeat receptor-like protein kinase family protein	−8.76	9.13 × 10^−33^	−8.33	2.81 × 10^−30^
	a,b,c	Lus10023323	AT5G46330	FLS2	Leucine-rich receptor-like protein kinase family protein	−5.86	2.27 × 10^−53^	−4.86	1.13 × 10^−44^
	a,b,c	Lus10021769	AT4G33300	ADR1-L1	ADR1-like 1	−5.6	7.13 × 10^−24^	−5.82	4.23 × 10^−23^
	a,b,c	Lus10034303	AT1G13340		Regulator of Vps4 activity in the MVB pathway protein	−5.21	3.82 × 10^−30^	−6.93	1.88 × 10^−36^
	a,b,c	Lus10036167	AT4G24580	REN1	Rho GTPase activation protein (RhoGAP) with PH domain	−4.37	4.35 × 10^−56^	−5.06	1.46 × 10^−59^
	a,b,c	Lus10040592	AT1G75820	CLV1,FAS3,FLO5	Leucine-rich receptor-like protein kinase family protein	−3.95	9.34 × 10^−11^	−4.05	6.10 × 10^−11^
	a,b,c	Lus10023879	AT2G36380	PDR6	pleiotropic drug resistance 6	−3.87	2.14 × 10^−3^	−4.67	6.74 × 10^−3^
	a,b,c	Lus10027196	AT5G62310	IRE	AGC (cAMP-dependent, cGMP-dependent and protein kinase C) kinase family protein	−3.34	1.85 × 10^−8^	−2.99	5.21 × 10^−7^
	a,b,c	Lus10023842	AT1G49470		Family of unknown function (DUF716)	−2.29	1.56 × 10^−17^	−1.76	5.73 × 10^−11^
	a,b,c	Lus10017267	AT1G75820	CLV1,FAS3,FLO5	Leucine-rich receptor-like protein kinase family protein	−2.16	1.86 × 10^−10^	−1.64	2.06 × 10^−6^
miR394	a,b	Lus10003801	AT3G22142		Bifunctional inhibitor/lipid-transfer protein/seed storage 2S albumin superfamily protein	−8.55	3.26 × 10^−7^	−5.32	1.77 × 10^−6^
	a,b	Lus10021450	AT5G60720		Protein of unknown function, DUF547	−6.8	2.36 × 10^−64^	−6.36	7.89 × 10^−64^
	a,b	Lus10039263	AT4G25640	DTX35,FFT	detoxifying efflux carrier 35	−5.64	3.15 × 10^−6^	−4.43	2.24 × 10^−5^
	a,b	Lus10033103	AT5G62720		Integral membrane HPP family protein	−3.81	1.63 × 10^−9^	−2.92	1.84 × 10^−6^
	a,b	Lus10020331	AT5G17540		HXXXD-type acyl-transferase family protein	−3.62	1.24 × 10^−3^	−7.21	6.10 × 10^−5^
	a,b	Lus10016114	AT5G60720		Protein of unknown function, DUF547	−3.15	8.12 × 10^−3^	−2.24	6.61 × 10^−2^
	a,b	Lus10028650	AT1G29470		S-adenosyl-L-methionine-dependent methyltransferases superfamily protein	−3.02	6.12 × 10^−68^	−2.82	3.44 × 10^−59^
	a,b	Lus10036563	AT4G00750		S-adenosyl-L-methionine-dependent methyltransferases superfamily protein	−2.82	6.15 × 10^−5^	−1.34	6.69 × 10^−2^
	a,b	Lus10017657	AT1G53210		sodium/calcium exchanger family protein/calcium-binding EF hand family protein	−2.75	1.21 × 10^−4^	−1.58	3.59 × 10^−2^
	a,b	Lus10010152	AT1G33170		S-adenosyl-L-methionine-dependent methyltransferases superfamily protein	−2.46	1.26 × 10^−14^	−1.45	3.31 × 10^−6^
	a,b	Lus10026325	AT3G18870		Mitochondrial transcription termination factor family protein	−2.23	6.14 × 10^−4^	−1.68	1.07 × 10^−2^
miR399	d	Lus10022548	AT3G54700	PHT1;7	phosphate transporter 1;7	−11.35	7.73 × 10^−13^	−11.15	3.19 × 10^−12^
	d	Lus10016821				−8.64	4.81 × 10^−6^	−5.97	1.33 × 10^−4^
	d	Lus10002217	AT5G54960	PDC2	pyruvate decarboxylase-2	−6.19	7.04 × 10^−54^	−5.15	4.97 × 10^−53^
	d	Lus10025530	AT3G59310		Eukaryotic protein of unknown function (DUF914)	−2.56	3.37 × 10^−27^	−2.2	1.61 × 10^−20^
	d	Lus10009312	AT2G45670		calcineurin B subunit-related	−2.07	8.47 × 10^−34^	−1.94	1.58 × 10^−29^

**Table 5:** List of genes up-regulated in the iFIBa sample compared to all other samples (a cutoff |log_2_FC| value iFIBa vs. iFIBb ≥ 1 with padj < 0.05, as well as an additional filter |log_2_FC| iFIBa&b vs. cPAR ≥ 1; TGR of iFIBa > TGR of any other sample). Log_2_FC values with padj < 0.05 are highlighted in bold. A heatmap displays normalized gene expression values in the corresponding samples.

Transcript ID	Description	At Homolog	At-Symbol	iFIBa vs. iFIBb, log_2_FC	iFIBa vs. tFIB, log_2_FC	iFIBa&b vs. cPAR, log_2_FC	cPAR	iFIBa	iFIBb	tFIB	sXYL
Lus10009510	Transducin/WD40 repeat-like superfamily protein	AT3G50390		**4.70**	**6.85**	1.05	5.8	22.5	0.8	0.0	17.5
Lus10016231	Disease resistance-responsive (dirigent-like protein) family protein	AT1G65870		**3.48**	**4.22**	**6.15**	0.6	98.4	8.8	5.3	2.0
Lus10038092	Myb domain protein 82	AT5G52600	MYB82	**3.06**	**9.59**	2.68	13.3	150.5	18.1	0.0	0.0
Lus10030235	Laccase 6	AT2G46570	LAC6	**2.17**	**9.55**	**3.95**	6.1	145.8	32.4	0.0	0.6
Lus10021514	Heparan-alpha-glucosaminide N-acetyltransferase-like protein (DUF1624)	AT5G27730		**2.06**	**3.15**	1.80	11.8	65.6	15.6	7.4	30.3
Lus10013125	Flavin-binding monooxygenase family protein	AT5G11320	YUC4	**1.97**	**9.35**	**5.62**	2.0	127.7	32.5	0.0	18.5
Lus10037136	Argonaute family protein	AT1G69440	AGO7,ZIP	**1.83**	**2.10**	**1.54**	85.5	387.6	109.2	90.7	178.8
Lus10020787	Acyl activating enzyme 12	AT1G65890	AAE12	**1.80**	**6.03**	**2.67**	70.3	691.2	197.8	10.6	3.9
Lus10015513	Glutamine dumper 3	AT5G57685	GDU3,LSB1	**1.75**	**2.19**	**3.21**	12.9	173.3	51.6	38.1	115.8
Lus10012546	Uncharacterized protein	AT3G01960		**1.57**	**7.56**	**6.45**	1.6	200.2	67.6	1.1	0.0
Lus10020352	Growth-regulating factor 5	AT3G13960	GRF5	**1.40**	**1.95**	**2.24**	39.9	271.5	103.1	70.5	56.8
Lus10015909	Basic helix-loop-helix (bHLH) DNA-binding superfamily protein	AT4G02590	UNE12	**1.34**	**3.38**	**5.13**	9.9	495.9	195.3	47.6	341.3
Lus10008497	AT hook motif DNA-binding family protein	AT1G63470		**1.31**	**2.94**	**2.46**	32.2	244.0	98.1	31.8	166.7
Lus10042938	Response regulator 7	AT1G19050	ARR7	**1.21**	**1.02**	**2.15**	24.1	145.0	62.6	71.5	54.7

## Supplementary Materials

The following are available online at http://www.mdpi.com/2223-7747/8/2/47/s1, Table S1: List of known flax miRNAs expressed in intrusively growing phloem fibers (iFIBa and iFIBb) compared with cortex parenchyma (cPAR), phloem fibers isolated at the stage of tertiary cell wall deposition (tFIB), and xylem (sXYL); Table S2. List of expressed flax genes in intrusively growing fibers (iFIBa and iFIBb) compared with cortex parenchyma (cPAR), phloem fibers isolated at the stage of tertiary cell wall deposition (tFIB) and xylem samples, enriched in secondary cell walls (sXYL) (a cutoff TGR > 16 in at least one sample); Table S3 List of differentially expressed genes in the iFIBa sample compared to the other samples analyzed (the cutoff |log_2_FC| values iFIBa vs. iFIBb > 1 with padj < 0.05). A heatmap displays the normalized gene expression values in the corresponding samples.
